# Linear Micro-patterned Drug Eluting Balloon (LMDEB) for Enhanced Endovascular Drug Delivery

**DOI:** 10.1038/s41598-018-21649-7

**Published:** 2018-03-05

**Authors:** KangJu Lee, Seul Gee Lee, Ilkwang Jang, Seung Hyun Park, Dasom Yang, Il Ho Seo, Sung-Kyung Bong, Duk Hwan An, Min Kwon Lee, In Kwon Jung, Yong Hoon Jang, Jung Sun Kim, WonHyoung Ryu

**Affiliations:** 1 School of Mechanical Engineering, Yonsei University, 50 Yonsei-ro, Seodaemun-gu, Seoul 03722 Republic of Korea; 2 Graduate Program in Science for Aging, Yonsei University, 50 Yonsei-ro, Seodaemun-gu, Seoul 03722 Republic of Korea; 3 Genoss Co., Ltd., 1F, Gyeonggi R&DB center/226 2F GSBC, 105 Gwanggyo-ro, Yeongtong-gu, Suwon-si 16229 Republic of Korea; 4 Division of Cardiology, Severance Cardiovascular Hospital, Yonsei University College of Medicine, 50 Yonsei-ro, Seodaemun-gu, Seoul 03722 Republic of Korea

## Abstract

In-stent restenosis (ISR) often occurs after applying drug eluting stents to the blood vessels suffering from atherosclerosis or thrombosis. For treatment of ISR, drug eluting balloons (DEB) have been developed to deliver anti-proliferative drugs to the lesions with ISR. However, there are still limitations of DEB such as low drug delivery efficiency and drug loss to blood flow. Although most researches have focused on alteration of drug formulation for more efficient drug delivery, there are few studies that have attempted to understand and utilize the contact modality of DEB drug delivery. Here, we developed a linear micro-patterned DEB (LMDEB) that applied higher contact pressure to enhance drug stamping to vascular tissue. *Ex vivo* and *in vivo* studies confirmed that higher contact pressure from micro-patterns increased the amount of drug delivered to the deeper regions of vessel. Finite element method simulation also showed significant increase of contact pressure between endothelium and micro-patterns. Quantitative analysis by high performance liquid chromatography indicated that LMDEBs delivered 2.3 times higher amount of drug to vascular tissue *in vivo* than conventional DEBs. Finally, efficacy studies using both atherosclerotic and ISR models demonstrated superior patency of diseased vessels treated with LMDEB compared to those treated with DEB.

## Introduction

To treat coronary or peripheral vascular disease, many advances have been made in the field of percutaneous coronary intervention, ranging from balloon angioplasty to drug eluting stent (DES)^1–3^. These technologies were developed mainly to reduce restenosis rates after interventions. Although DES has been a standard treatment for patients with vascular occlusion or thrombosis^4,5^, in-stent restenosis (ISR) often followed stent treatment as an inevitable adverse consequence^6^.

Coating of polymeric matrix containing anti-proliferative drug on the surface of DES significantly reduces ISR occurrence. However, the degradation and endocytosis of the coating formulation of DES often induce inflammation and thrombosis that can lead to restenosis^7^. There have been a number of treatments for ISR including balloon angioplasty, implantation of a second stent, rotablation, and brachytherapy^8–12^. However, since the first clinical trial of drug eluting balloon (DEB) for treatment of ISR in human^13^, DEB has shown encouraging outcomes and is recommended as a guideline against ISR from prominent societies of medical professions^14,15^.

In recent years, formulations of drug coating of DEBs have been extensively investigated for efficient delivery of an anti-proliferative drug, paclitaxel. The most representative coating methods for DEB are PACCOCATH® (Oberpfaffenhfen, Germany) and DIOR® (Bonn, Germany) technology^16–18^. The PACCOCATH® uses iopromide as a hydrophilic spacer coating that has porous structure with high contact efficiency between the vessel lesion and the lipophilic paclitaxel (PTX)^16^. DIOR® has a coating layer of a mixture of PTX and shellac on the roughened surface of a DEB without matrix polymer. This characteristic achieves higher bioavailability of the paclitaxel and efficient delivery to the lesion upon inflation^16,19^. For enhanced local drug delivery, balloon structures were also modified using double balloons or porous surfaces^20,21^.

There, however, are still common limitations of the current DEB technologies relating endovascular drug delivery efficiency^22,23^. For sufficient release of drug, DEB dilation is maintained for 60 seconds to make a contact with a vascular lesion. Due to this prolonged blockage of vessels, the use of DEB is not recommended for patients who are vulnerable to additional complications. More importantly, although DEB drug delivery depends only on a short contact between a drug-coated balloon and a vascular lesion, the drug transfer mechanism is poorly understood. The drug delivery efficiency of a DEB is generally low, and more systematic investigation is necessary to understand how drug molecules are transferred from a DEB surface to target tissue lesion during their contact. Loss of drug during balloon handling and insertion through catheters to the target lesion is also significant. The coated drug can also be washed off of the DEB surfaces due to a highly viscous blood stream.

The fundamentals of drug adsorption from DEB to lumen surface are based on a combination of multiple modes of interactions such as electrostatic interaction, van der Waals force, and entropic effects between drug coating and endothelium on a molecular scale^24,25^. Thus, most DEB studies to enhance the efficiency of drug adhesion have relied on methods such as modulation of hydrophilic drug carriers for delivering lipophilic anti-proliferative drugs to the lesion lumen^13–17,19^. However, a firm contact between the surface of inflated balloon and lumen is a critical prerequisite for sufficient drug adsorption onto the target tissue in the lumen surface to occur. Compared to the flat surface of conventional DEBs, it is anticipated that the use of micro-patterned surface may provide a firm contact and increase a contact pressure in particular for soft and deformable tissue like blood vessels. Although such enhanced contact and higher pressure from micro-patterned DEBs are likely to increase the efficiency of drug delivery, there has been no attempt of investigating the effect of micro-patterned DEBs yet.

**Figure 1:**
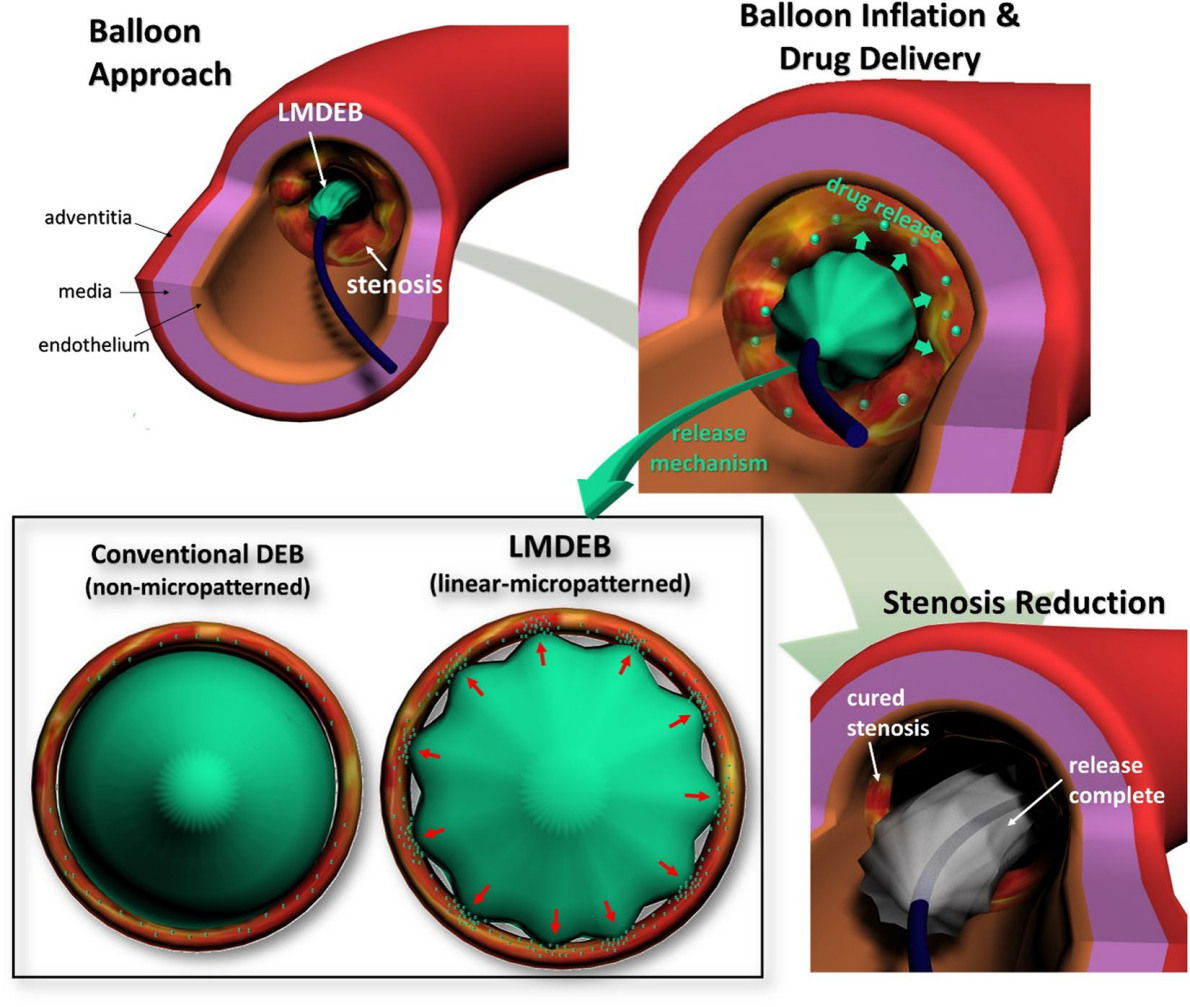
Schematic of linear micro-patterned drug eluting balloon (LMDEB) application to the stenosis lesion of blood vessel. Enhancement of drug delivery by increasing contact pressure at micro-patterns after full inflation.

Herein, we propose a linearly micro-patterned drug eluting balloon (LMDEB) to enhance the efficiency and accuracy of endovascular drug delivery to a target lesion by increasing contact pressure between the coating and inner vessel wall. LMDEBs were fabricated using a commercial manufacturing balloon forming machine based on a blow molding process with micro-machined balloon forming molds. Prior to conventional balloon angioplasty with DEB for endovascular drug delivery, the diameter of lesion vessel is inflated 1.3 times using a non-coated balloon catheter, which is a standard procedure for any DEB intervention. Then, as shown in Fig. 1, LMDEB becomes inflated up to a size equivalent to the enlarged diameter of the pre-stretched lesion vessel. During this inflation, the LMDEB forms more localized contacts with the luminal side of the vascular tissue. This induces higher contact pressure and more efficient “drug stamping” on the target lesion than DEBs without micro-patterns.

## Results

### Fabrication of LMDEB

**Figure 2:**
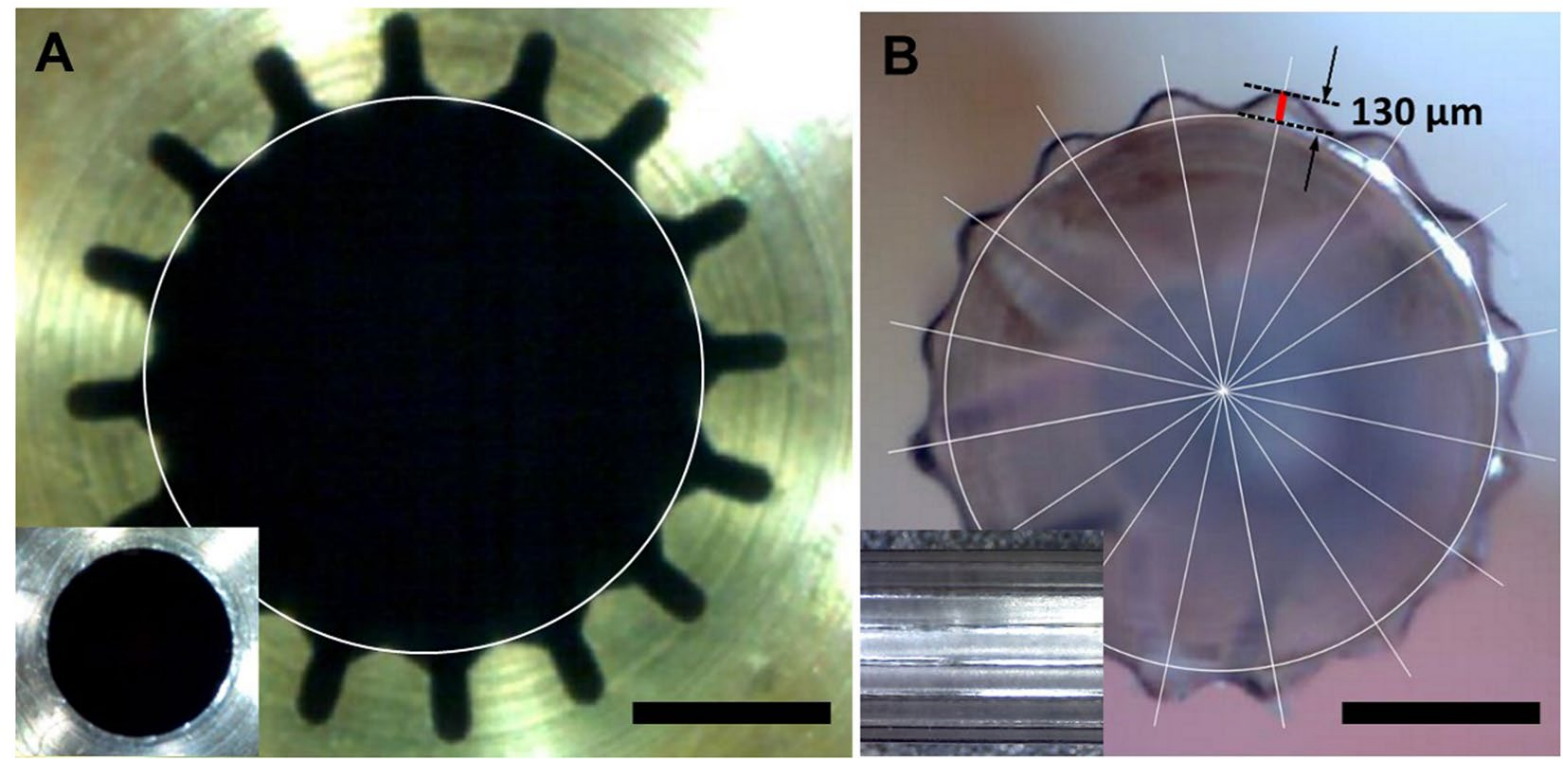
(**A**) Cross-sectional view of a micro-machined mold with an inset showing a non-machined mold for conventional drug eluting balloons (DEBs). (**B**) Cross-sectional view of LMDEB when fully inflated. The LMDEB has 16 conformal linear micro-patterns of 130 µm height with an inset showing lateral view. All scale bars indicate 1 mm.

**Figure 3:**
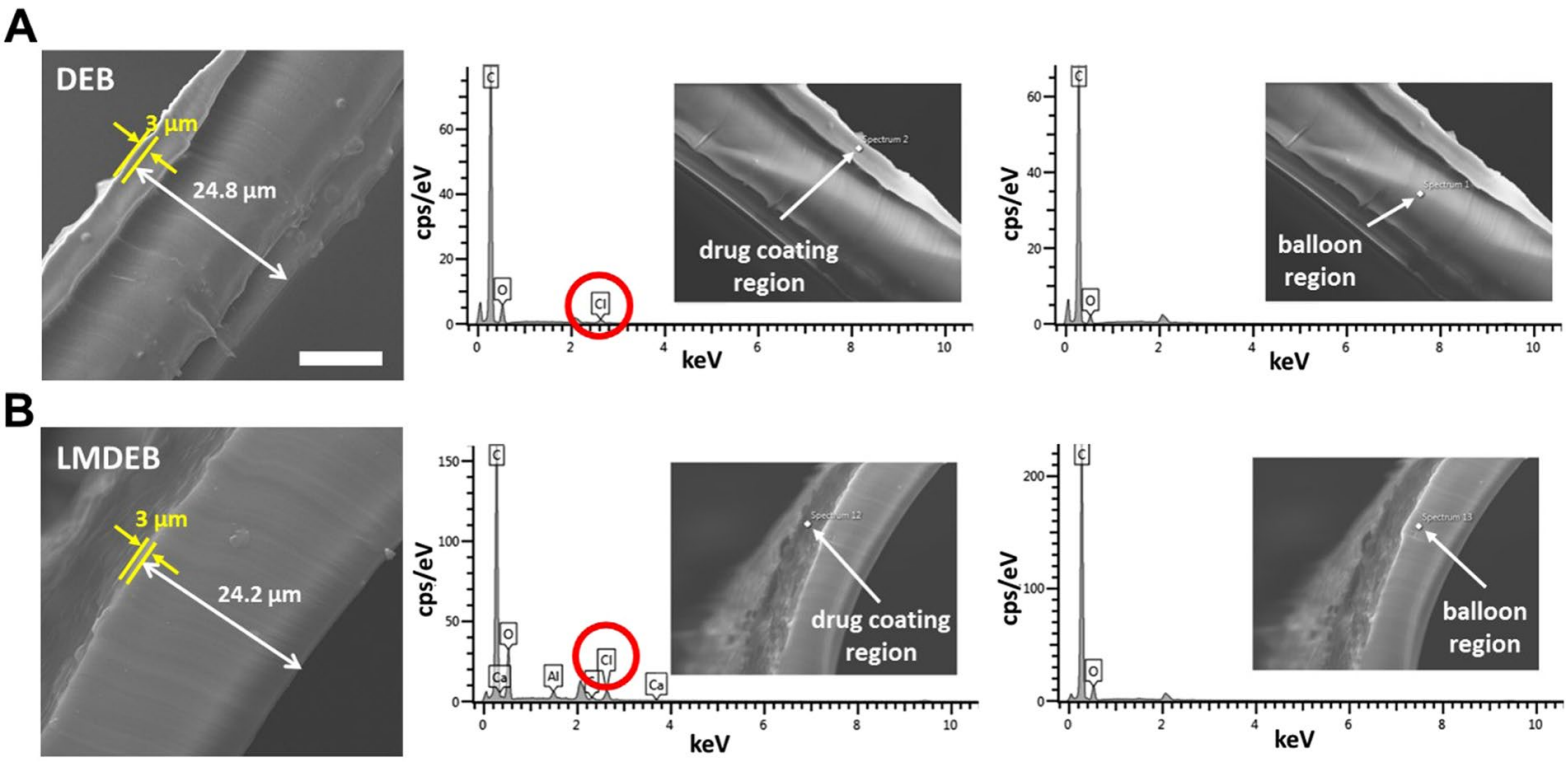
Drug coating of (**A**) DEB and (**B**) LMDEB. Both thicknesses of drug coating and formed of either LMDEB or DEB are identical to about 3 µm and 25 µm. All scale bars indicate 10 µm.

LMDEBs were fabricated by the following process; thin cylinder tubes made of polyether block amide elastomer (PEBAX) were blow-molded in a micro-machined balloon forming mold. The micro-machined mold was fabricated by an electric discharge machining (EDM) wire-cut process to have a linear array of intaglio linear micro-patterns (LMs) on the inner surface of the mold. The widths and intervals of the LMs were determined by the limitation of EDM wire-cut process for mold fabrication. We hypothesized that wider widths and intervals would decrease the contact pressure. Therefore, the width and intervals were the smallest that could be fabricated by the EDM process, in an effort to increase contact pressure (in Fig. 2A). We also hypothesized that taller micropatterns would be more beneficial in achieving focused contact with the endothelium. Therefore, the height (130 μm) of the micropatterns with round tips at uniform intervals of 22.5° was the tallest that we could achieve from our balloon molding process as shown in Fig. 2B. The thickness of the finished LMDEB was about 25 µm, and the dimension of LMDEB was 2.75 mm (diameter) × 10 mm (length). The dimensions were pertinent to be deployed at rabbit iliac artery. The amount of drug coating was 260 µg equally for both DEB and LMDEB. The drug was uniformly coated with 3 µm thickness. The coating thickness was confirmed by energy dispersive x-ray spectroscopy (EDS) analysis by tracking chlorine (Cl) ion, which exists only in paclitaxel coating matrix (Fig. 3). LMDEB was uniformly coated with drug along both the smooth and micro-patterned profiles of LMDEB surface. Pleating and folding processes were performed to prepare LMDEBs for their interventional application by reducing balloon profile.

### Dependency of drug stamping on contact pressures

**Figure 4:**
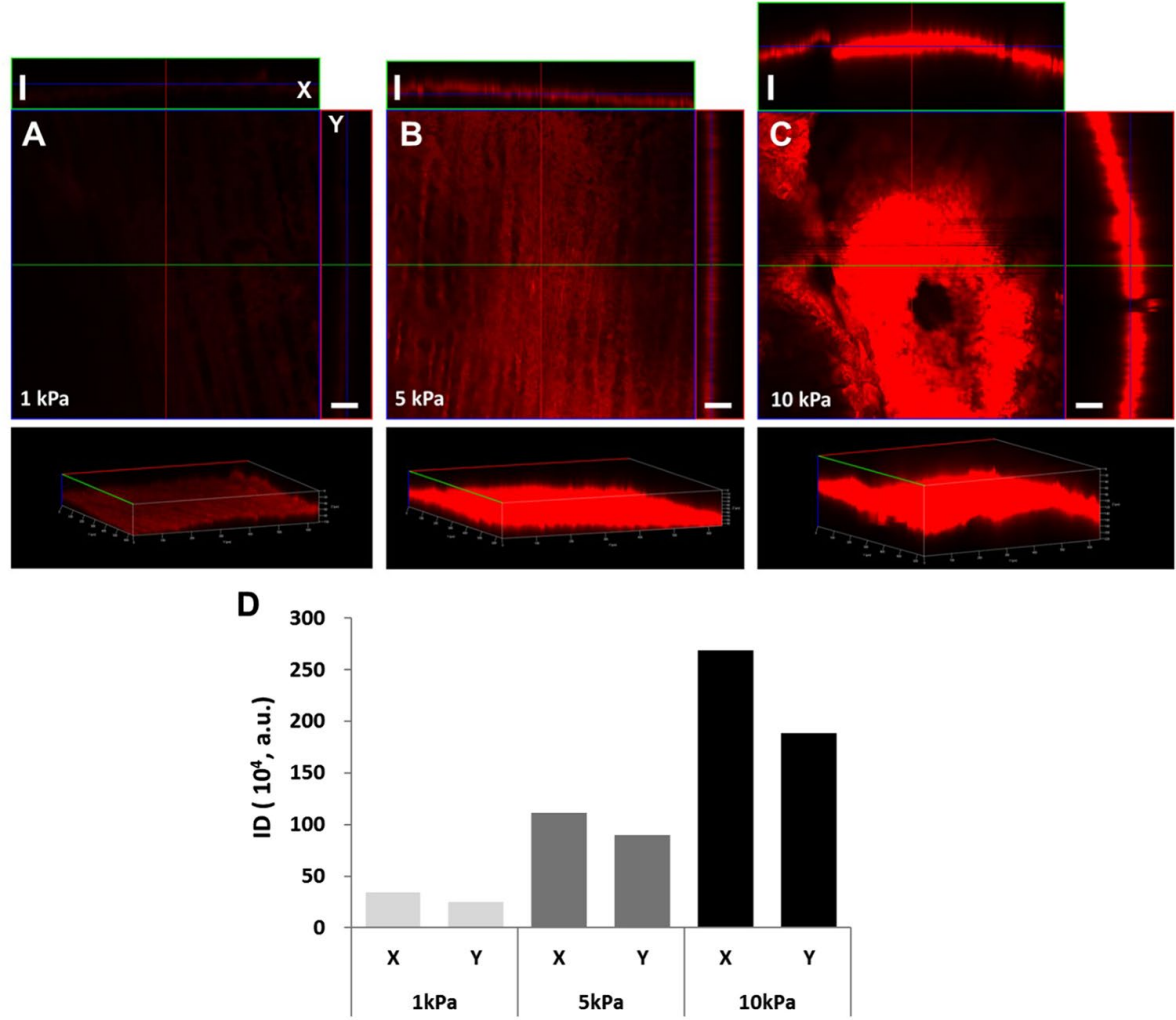
Dependency of drug stamping on contact pressures. Laser scanning fluorescent confocal image of tissue samples stamped under contact pressure of (**A**) 1 kPa, (**B**) 5 kPa, and (**C**) 10 kPa. (**D**) Comparative plot of integrated density (ID) from *ex vivo* tissues with contact pressure applied in range of 1 to 10 kPa. All scale bars indicate 50 µm.

To prove our hypothesis that higher contact pressure enhances drug transfer from a DEB surface to endothelium, *ex vivo* tests were performed with harvested fresh endothelium samples. RB-coated DEB stamps were prepared and they were stamped on the endothelium tissue samples under contact pressures ranging from 1 to 10 kPa. Then, the distribution and the degree of infiltration of RB within *ex vivo* tissues were analyzed by confocal microscopic analysis. The fluorescent RB signals from each stamped tissue sample were quantified using the NIH ImageJ program by introducing integrated density (ID). The ID values were calculated from the fluorescent drug signals to quantify the drug distribution in the stamped endothelium tissue samples. We measured the ID of each sample from X- and Y-axis projection images of Fig. 4A–C. The fluorescent RB signal was stronger for the samples stamped under higher contact pressures (Fig. 4D). This indicates a strong correlation between the amount of drug delivered to the tissue samples and the contact pressure. Interestingly, the depth of drug dispersion into the tissue sample also showed strong dependency on the contact pressure. The samples exposed to the contact pressure of 1 kPa showed a shallow depth of drug dispersion (below 10 µm), while the drug molecules reached down to the depths of about 20 and 50 µm, respectively, in the samples exposed to 5 and 10 kPa. This result indicates the direct dependency of drug delivery efficiency on the contact pressure between the drug-coated DEB surfaces and vascular tissue.

### Contact pressure in vascular tissue surface by LMDEB vs. DEB

**Figure 5:**
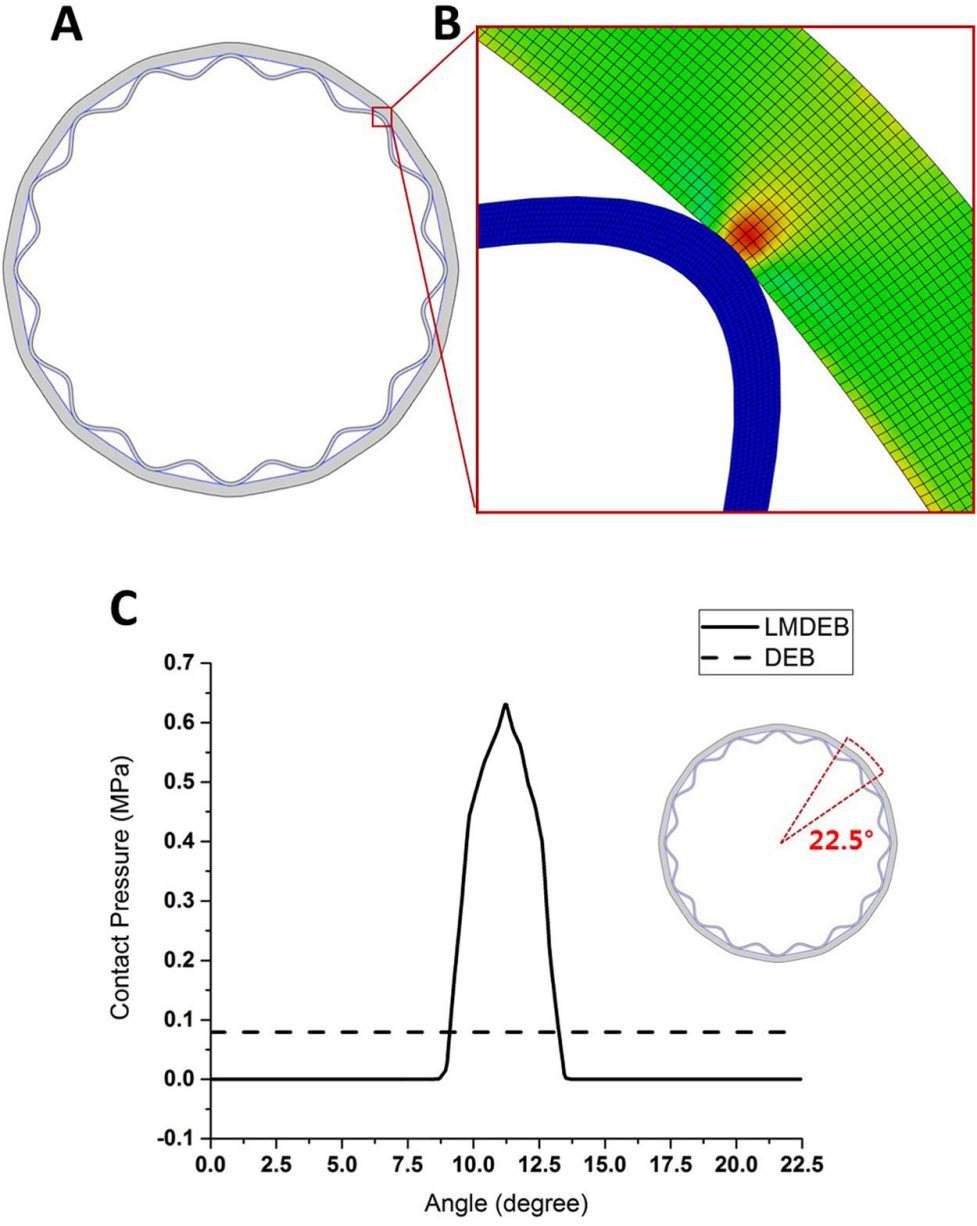
(**A**) Contact interaction of LMDEB and vascular tissue when LMDEB is fully expanded to the diameter of 2.75 mm. (**B**) Finite element model in a contact region. Red color indicates higher stress. (**C**) Contact pressure distribution along the artery surface of the cyclic symmetry section ranging from 0 to 22.5 degree.

In order to further confirm our hypothesis, we conducted a finite element method (FEM) analysis to compare the contact pressures generated by LMDEB and conventional DEB in contact with the luminal vessel wall by simulating the environment of balloon inflation drug intervention. As shown in Fig. 5A and B, the contact region of vascular tissue has higher stress values (red color indicates higher stress) than other regions. Figure 5C shows the contact pressure distributions of the cyclic symmetry section, ranging from 0 to 22.5 degree, for the two different cases. If the diameters of DEB and LMDEB increase up to 2.75 mm, the maximum contact pressures at the artery surface increase up to 0.079 MPa and 0.631 MPa for DEB and LMDEB, respectively. The maximum contact pressure of LMDEB is eight times higher than that of DEB.

In this FEM analysis, the LMDEB and DEB are assumed to be inflated uniformly in a radial direction. However, the inflation process of the balloons in clinical situations is slightly different from the simulated process. In a clinical situation, a DEB is folded and rolled up to be inserted into a catheter, and it is delivered to a target lesion through tortuous blood vessels. Then, the balloon is unrolled and unfolded during balloon inflation. The inflation process was omitted for the FEM simulation since the inflation took only a couple of seconds, compared to the contact time of 60 seconds. Thus, the FEM analysis focused on the final contact step for 60 seconds for drug delivery instead of modeling the entire process of balloon inflation.

### Drug distribution in vascular tissue by LMDEB vs. DEB

In order to confirm enhanced drug delivery of LMDEBs that can apply higher contact pressure than DEBs with flat surfaces, *in vivo* studies were performed on the left and right iliac arteries of rabbits using LMDEBs and DEBs. They were coated with model drugs, rhodamine B (RB) (n = 7) or a mixture of fluorophore (FP)-labeled PTX and pure PTX, PTX + FP, (n = 5). The formulation of PTX + FP was especially used in this study for more accurate prediction of drug distribution within the tissue by eliminating the property difference between RB and PTX. LMDEB and DEB interventions were performed at target sites, and no embolism, which could be induced by the fragments of drug coating, was observed throughout the entire study. After the interventions with the DEBs and LMDEBs, the treated vessels were harvested after 48 hours. The fluorescent signals of the model drugs were visualized after cryo-sectioning the harvested samples to preserve the distribution of the fluorescent signals, which are significantly altered during a long preparation process of paraffin-sectioning (Supplementary Data Figure S1).

**Figure 6:**
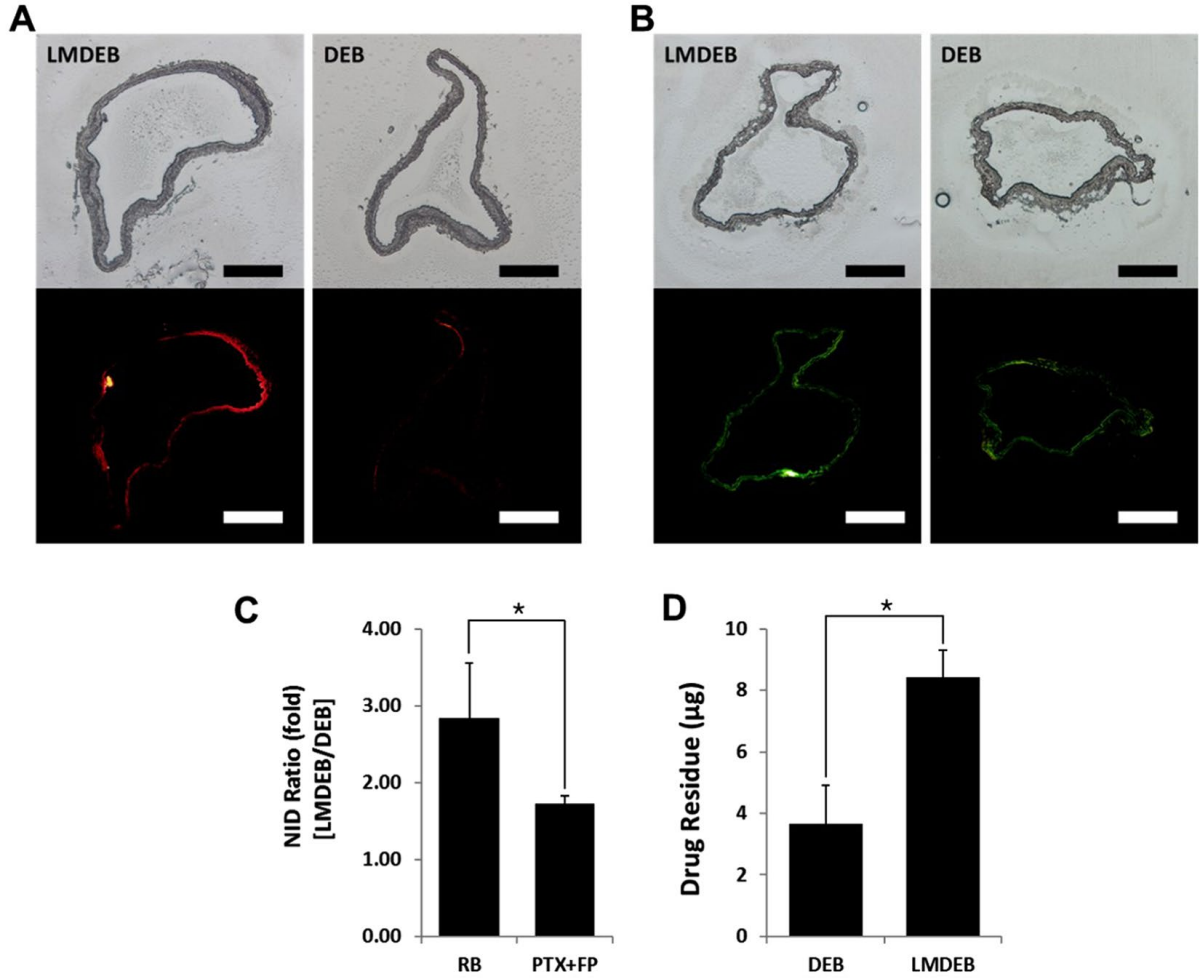
(**A**) Microscopic images of rhodamine B (RB) distribution in vessels treated with LMDEB and DEB. Upper images are unstained optical images and below images are the corresponding fluorescent images. (**B**) Microscopic images of paclitaxel conjugated green fluorophore (PTX + FP) distribution in treated vessels treated with LMDEB and DEB. Upper images are unstained optical images and below images are the corresponding fluorescent images. (**C**) Normalized integrated density (NID) ratios of LMDEB/DEB for RB (n = 7) and PTX + FP (n = 5). *p < 0.001. (**D**) Residual amount of PTX remained in vessels treated with DEB and LMDEB (n = 5). All scale bars indicate 500 µm. *p < 0.05, compared with DEB group. All data were presented as the mean ± SEM.

As shown in Fig. 6A and B, fluorescent microscopic analysis was performed to detect drug distribution within vascular tissue treated with model drugs. All the conditions of image acquisition such as light intensity (6), exposure time (1 second), and sensitivity to light (ISO 100) were maintained identical throughout the analysis. For both RB and PTX + FP coatings, LMDEB treated samples showed higher fluorescent signals of the model drugs than DEB treated samples. This result demonstrates that drug can be delivered more effectively to the endovascular tissue by LMDEBs than DEBs. To compare and analyze the drug distribution quantitatively, the ID of each fluorescent image was measured and then normalized by each sectional area of the vessel sample at the pixel scale. The normalized integrated densities (NIDs) of LMDEB (left artery) and DEB (right artery) tissue samples were compared with each other, as shown in Fig. 6C. It is shown that LMDEB has 2.84 (±0.71) times higher NID of RB than DEB, while LMDEB has 1.73 (±0.09) times higher NID of PTX + FP than DEB.

### Quantification of drug amount transferred to vascular tissue

To quantitatively assess drug amount delivered to target vascular tissue, the amounts of drug residue within the vessels were measured by tissue homogenization and liquid chromatography mass spectrophotometry (LC/MS) analysis. The left (LMDEB) and right (DEB) iliac arterial tissues from 5 rabbits were harvested after 48 hours following balloon dilatation, and then the cultured tissues were homogenized and filtered in the liquid form. The PTX amounts in these liquid samples were quantified by LC/MS analysis. The result (Fig. 6D) indicates that DEB treated tissues retained 3.67 (±1.24) µg of PTX on average, while LMDEB treated tissues retained 8.43 (±0.89) µg of PTX. The detected amounts of PTX retained in tissue after 48 hours of balloon inflation are both 1.4% and 3.2% of drug coated on the surfaces of DEBs and LMDEBs, respectively. These results strongly support that LMDEBs transfer drug more efficiently to vascular tissue than DEBs with flat surfaces.

### Therapeutic effect of LMDEB vs. DEB in atherosclerosis model

To demonstrate the enhanced efficacy of LMDEB, we performed *in vivo* tests with a vascular disease model. An atherosclerosis model was employed using rabbits whose iliac arteries were balloon injured after fed a high fat diet for five weeks. Balloon injured rabbits were treated under the same condition as described above. Both DEBs and LMDEBs were coated with 260 µg of PTX. There were four groups in this study as follows: negative control group (normal rabbit, n = 4), positive control group (atherosclerosis model, n = 8), LMDEB treated group (atherosclerosis model, n = 6), DEB treated group (atherosclerosis model, n = 6). Quantitative coronary angiography (QCA) and optical coherence tomography (OCT) were performed immediately after balloon inflation and after 4 weeks. The progress of intimal formation was quantitatively analyzed in terms of diameter stenosis (DS) and area stenosis (AS). After the 4-week follow-up study, all vascular tissues were harvested and stained with trichrome and hematoxylin and eosin (H&E) for histological analysis to analyze vascular integrity and intimal formation.

**Figure 7:**
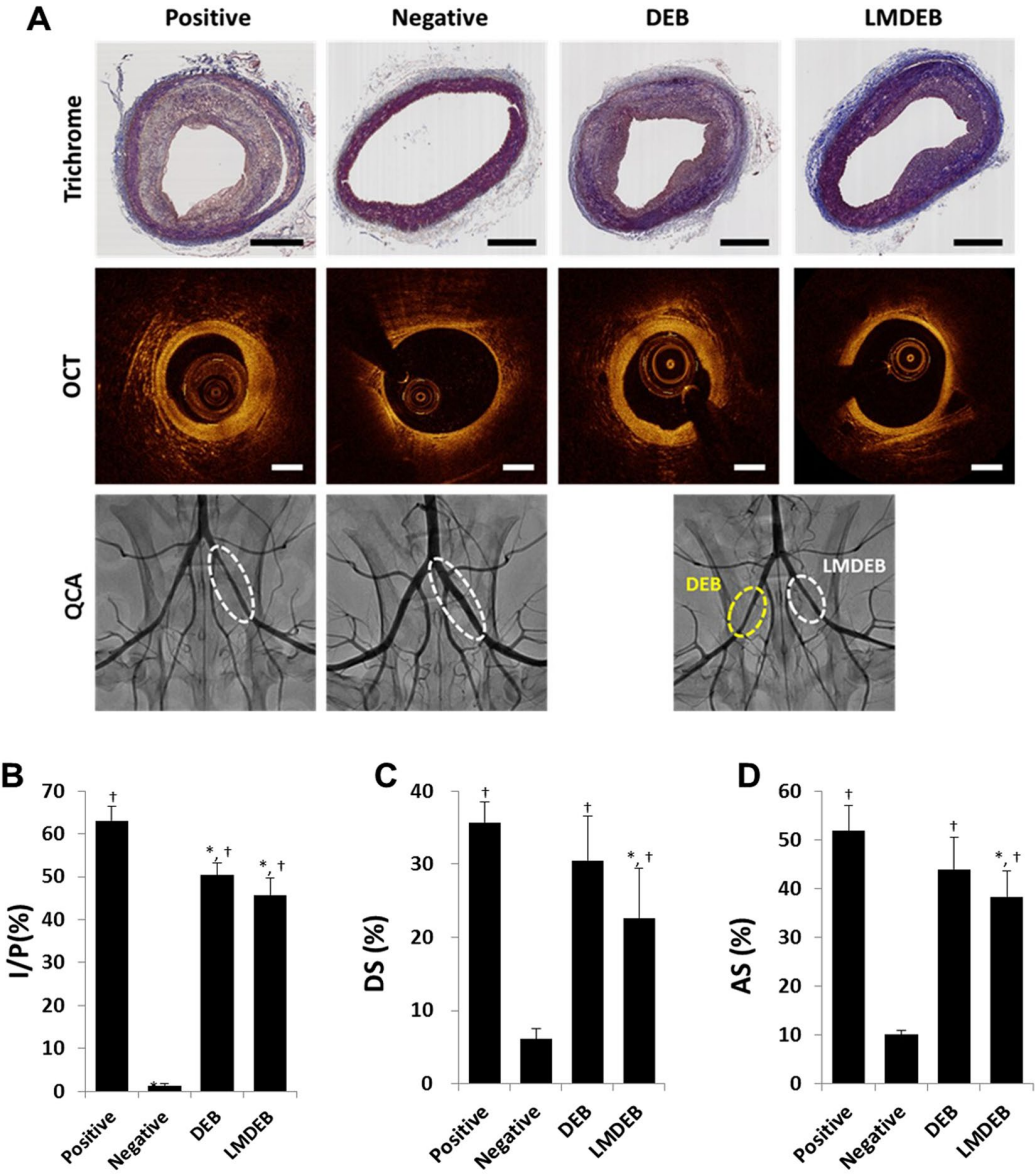
(**A**) Histopathological (trichrome stained), optical coherence tomography (OCT), and quantitative coronary angiography (QCA) images of vessels treated with positive, negative, DEB, and LMDEB. The samples were stained with trichrome. All scale bars indicate 500 µm. (**B**) Intima to plaque ratios of positive group: 63.04 ± 3.39%, negative group: 1.31 ± 0.57%, DEB group: 50.4 ± 2.86% and LMDEB group: 45.58 ± 4.19%.(p = 0.001 by ANOVA), (**C**) Percentage of diameter stenosis (DS) of positive group (n = 4): 35.7 ± 2.81%, negative group (n = 8): 6.10 ± 1.37%, DEB group (n = 6): 30.47 ± 6.16%, and LMDEB group (n = 6): 22.65 ± 6.77% (p = 0.002 by ANOVA), (**D**) Percentage of area stenosis (AS) of positive group: 51.78 ± 5.27%, negative group: 10.08 ± 0.91%, DEB group: 43.95 ± 6.60% and LMDEB group: 38.33 ± 5.32% (p = 0.007 by ANOVA). All data were presented as the mean ± SEM. *p < 0.05, compared with positive group. ^†^p < 0.05, compared with negative group.

First, the degree of intimal formation was investigated from proximal, mid, to distal sections at 3.3 mm intervals. Significant intima proliferation was observed in the positive control group, as expected (Fig. 7A). The stenosis progress in each group was quantified by measuring medial area, intimal area, and plaque area as follows: 1) media = external elastic lamina − internal elastic lamina, 2) intima = internal elastic lamina − lumen, 3) plaque = media + intima. Then intima-plaque ratio (I/P), which indicates the degree of intimal formation with respect to the thickness of a normal media layer, was calculated. As shown in Fig. 7B, intimal layer thickness was significantly decreased in both DEB and LMDEB treated groups than in the positive control group.

DS and AS values were estimated from quantitative angiography and OCT analysis by detecting the whole length of vessel lesion, where PTX was delivered (Fig. 7A). Mean radial diameter (RD) and minimal lumen diameter (MLD) were calculated with QCA. DS was defined as the following formulation: DS (%) = (mean RD − MLD)/mean RD × 100. In Fig. 7C, the DS of the LMDEB group substantially decreased compared to both positive and DEB groups. In particular, there was a statistically-significant difference between LMDEB and positive control groups. Although the DEB group had smaller DS values than the positive group, there was no statistical significance. On the other hand, the areas of lumen, plaque, and vessel were calculated by OCT image analysis for estimation of AS values for each group. The AS value was derived with the following formula: AS (%) = (vessel − lumen)/vessel × 100. As indicated in Fig. 7D, the AS of the LMDEB group was also smaller than both positive and DEB groups. In particular, there was a statistically significant difference between LMDEB and positive groups. However, no statistically significant difference was found between positive and DEB groups. (Detailed calculation of the experimentally measured values is given in Supplementary Data Figure S2).

### Therapeutic effect of LMDEB vs. DEB in ISR model

We further investigated the therapeutic efficacy of LMDEB using an ISR model in minipigs. The ISR model was established by 4-week follow-up after stent implantation, and the ISR presence was confirmed before ballooning with LMDEB or conventional DEB. The drug amounts and the dimensions of both LMDEB and conventional DEB were kept the same as when they were applied to the atherosclerotic model. Three devices of both LMDEBs and DEBs were used for drug delivery in total 6 ISR lesions. After 4 weeks, DS and AS were measured in each lesion by QCA and OCT analysis to determine the degree of lesion reduction.

**Figure 8:**
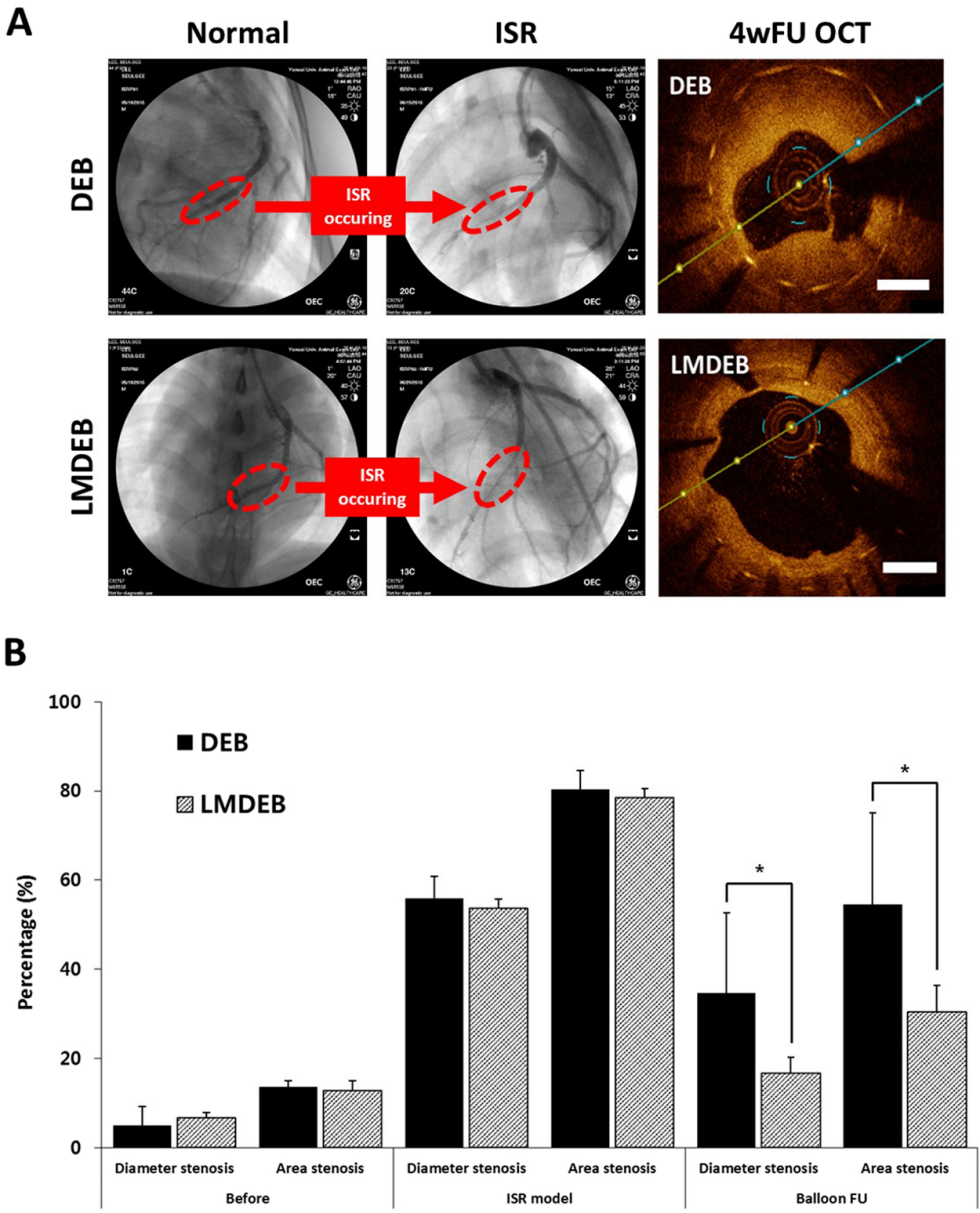
(**A**) QCA image indicating in-stent restenosis (ISR) modeling in minipig after 4 weeks stent implantation (4 insets on the left) and OCT image indicating therapeutic effect for ISR after 4 weeks balloon angioplasty (2 insets on the right). Scale bar indicates 1 mm. (**B**) Percentage of DS and AS of DEB (n = 3) and LMDEB (n = 3) groups; 34.67 ± 17.95% (DS, DEB group), 16.66 ± 3.56% (DS, LMDEB group), 60.39 ± 15.76% (AS, DEB group), 43.14 ± 6.40% (AS, LMDEB group). All data were presented as the mean ± SEM. * p < 0.05, compared with DEB group.

As shown in Fig. 8A, ISR formed in all blood vessels of minipigs, and these lesions were treated with either LMDEBs or conventional DEBs. After 4 weeks, the ISR lesion treated with LMDEB was significantly reduced at the lumen side compared to the lesion treated with the conventional DEB (Fig. 8B). Both DS and AS of LMDEB group (n = 3) had smaller values than the DEB (n = 3) group with statistical significance. It is noteworthy that the LMDEB group had statistically predominant reduction in DS and AS compared to the DEB group. (Detailed calculation of the experimentally measured values is given in Supplementary Data Figure S3).

## Discussions

Efficient drug delivery by DEBs has been reported to substantially reduce the occurrence rate of ISR^16,19^. DEB technologies have evolved rapidly by developing coating formulations or surface modification of DEBs for enhanced drug delivery. In addition, new drugs such as sirolimus or zotarolimus were introduced and their improved bioavailability and efficacy were demonstrated^26,27^. Different types of drug formulations to coat such limus-family drugs have also been developed using biodegradable polymer or nanoparticles^28–30^. However, this coating formulation-based approach strongly depends on the chemical properties of drugs, such as molecular weight or hydrophobicity, or on the properties of matrix materials used in the coating formulation. Although these characteristics of drug or drug formulation are likely to affect the drug retention rate within tissue, there are few studies that quantified the amount of drug residue so far. Furthermore, despite these rapid advances in drug coating/formulation as well as introduction of new drugs, DEB still suffers from significant drug loss during handling and application within vessels. Reduction in operation time of DEB application is also highly desired to minimize any side effect since blood flow is blocked during DEB treatments.

In this work, we demonstrated that higher contact pressure by micro-patterned DEBs enhanced transfer efficiency of drug compounds to vascular tissue without changing drug coating/formulation. The mechanism of material transfer from a surface to another based on their contact has been studied for a long time. For example, the effect of contact speed and pressure on the quality and precision of printing were well described in the ink printing technology for newsprints. Increasing contact (printing) pressure increased the amount of transferred ink, whereas increased printing speed decreased the total transfer^31^. It was also reported that contact pressure needed to be above a certain level for direct contact between ink and a substrate depending on ink viscosity^32^. Mechanisms for material transfer between an ink and a substrate have also been explained by capillary immobilization^33^ and ink transfer equation^34^. Since the first introduction of micro contact printing^35^, state-of-the art contact transfer technologies have been used in conventional gravure or letter printing as well as in metallic or organic electrode printing for flexible electrode^36^ or even protein printing for surface modification^37^. When it comes to development of advanced contact printing or transferring processes, the major factors are electrical and chemical surface characteristics of both stamp and ink for contact process as well as the ink and substrate of contact printing. However, moderate contact or impression is the precondition. In case of DEB development, there have been many studies for developing drug formulations with respect to their chemical and material characteristics, but no study was performed to utilize contact pressure for enhanced drug delivery yet.

It is hypothesized that LMDEBs have “higher contact pressure after balloon inflation” for endovascular drug delivery compared to conventional DEBs. A conventional DEB expands and makes a uniform contact to the pre-stretched vessel diameter. On the other hand, the narrow ridges of a LMDEB make more localized contacts with the luminal side of the vessels. As confirmed in the FEM analysis results (Figs 1 and 5), this results in higher contact pressure to the contact regions, and this leads to more efficient drug delivery to vascular tissue. It was also shown that higher contact pressure led to deeper infiltration of drug from the endothelium to the media layer of vascular tissue. Since blood flow is highly viscous and fast, such deeper drug delivery into the target tissue can minimize any potential loss of drug to blood streams. This deeper drug delivery to vascular tissue at each LM, in turn, increases the retention rate of drug and lengthens the duration of the efficacy.

With regard to the surface area of both DEBs and LMDEBs, the drug coated area of LMDEB (1 cm^2^) is 1.16 times larger than that of conventional DEB (0.86 cm^2^). This increased surface area of LMDEBs can also contribute to the enhanced delivery of drug molecules to the vascular tissue. However, since more than 200% higher amount of residual PTX was measured in LMDEB-treated tissue samples (Fig. 6D), the contribution of the increased surface area to the enhanced drug delivery is relatively small compared to the contribution of the increased contact pressure. Moreover, when blood vessel is fully stretched, actual contact between an LMDEB and the vascular tissue is likely to be limited to the small ridge area of the LMDEBs. This indicates that the increased surface area of LMDEB is not a major factor for enhanced drug delivery of LMDEB. On the other hand, the FEM analysis results showed 8 times higher contact pressure of LMDEBs than DEBs. Although further experimentation and analysis need to be performed with regard to the effect of the contact pressure and the surface area, this strongly suggests the main enhancement is originated from the higher contact pressure rather than the increased surface area of LMDEBs. Diseased vessels with ISR have non-uniform mechanical properties within the lesion due to the progress of stenosis and stents embedded at the site, which could be another factor in preventing uniform and reliable contact of DEB. The outcomes from our *in vivo* studies indicated that LMDEBs had enhanced delivery efficiency and therapeutic efficacy than DEBs, and this can be ascribed to deeper drug delivery by higher contact pressure as well as more uniform and reliable contacts even with ISR tissue.

We have developed LMDEB for enhanced drug delivery for ISR treatment. Ridge-shaped linear micro-patterns were formed on the surface of medical balloons by a blow-molding process for higher contact pressure during balloon inflation. The result of FEM analysis confirmed that the contact pressure of LMDEBs is higher than that of DEBs without any pattern. *Ex vivo* drug stamping tests also showed that higher contact pressure enhanced drug delivery to the luminal surface of blood vessels. *In vivo* studies with fluorescent and LC/MS analyses confirmed significantly higher drug delivery efficiency of LMDEBs than DEBs. Finally, *in vivo* efficacy tests were performed using atherosclerotic rabbit and ISR minipig models, and demonstrated statistically-significant therapeutic efficacy of LMDEBs compared to conventional DEB of LMDEBs.

## Methods

### Molding process for LMDEB and drug coating

LMDEBs were fabricated using a blow-molding process identical to a conventional DEB manufacturing process with customized balloon forming molds. Brass balloon forming molds were 10 mm long with the diameter of 2.75 mm. They had 16 intaglii LMs (300 µm depth) that were patterned the inside of a cylindrical mold by EDM wire-cut (Fig. 2A). The finished custom molds were installed in a balloon forming machine. A PEBAX cylinder tube (inner diameter: 0.60 mm, outer diameter: 1.00 mm) (Arkema, France) was placed in alignment to the center axis of the mold. The PEBAX tube was heated up to 130 °C and high pressure Ar gas at 30 bars was injected into the heated tube such that the tube expanded and formed a balloon. Upon completion of the molding process, the balloon and mold were rapidly cooled down to room temperature.

Then, the fabricated LMDEBs were folded to have three wings and flow-coated with a pipette using natural hydrophilic polymer coating formulation similar to PACCOCATH technology. RB (R6626, Sigma Aldrich, USA), PTX (P9600, LC Laboratories, USA) and the mixture of PTX labeled with a fluorescent molecule, Oregon Green 488 (P22310, Molecular Probes, USA), and pure PTX (w/w = 1:1000) (PTX + FP) were used for drug coating in this study. Finally, the drug coated balloons were assembled with catheter sheath and rolled up to reduce the balloon profile. After drug coating of the balloons, the cross-section of the balloon was examined to measure the thickness and uniformity of drug coating by tracking chlorine using an EDS equipped scanning electron microscope (JEOL 7800 F, JEOL, USA). The EDS analysis was carried out at ×2000 magnification and under 15 kV accelerated voltage.

### Effect of contract pressure on drug stamping

To understand how contact pressure affects the delivery efficiency of drug from DEB surfaces to vascular tissue, we performed *ex vivo* drug stamping test by varying contact pressures from 1 to 10 kPa. A portion of a DEB coated with RB was cut and attached to the bottom side of a square-shaped metal bar (4 × 4 mm^2^). This RB-coated DEB stamp was installed to an auto micro-stage for precise control of drug stamping. Harvested samples of rabbit femoral artery were fixed on a load cell with its lumen side up to have a direct contact with the stamp. Contact pressures of 1, 5, and 10 kPa were applied to each artery sample for 60 seconds. Then, these samples were stored in a sealed chamber under 100% humidity at 4 °C for 12 hours. The amount and distribution of the model drug, RB, within each artery tissue sample, was assessed using a fluorescent confocal microscope (LSM700, Zeiss, Germany). The pictures of all samples were taken under identical imaging conditions (exposure time, gain, filter). The 3D images for overall drug distribution and 2D orthogonal images from x- and y-axis projection for precise depth of drug infiltration were visualized by an image analyzer program (Zen 2011, Zeiss, Germany). To analyze drug distribution in the vascular tissue, the IDs of delivered RB within the tissue were quantitatively analyzed using ImageJ software (National Institutes of Health, USA) from the 2D orthogonal images according to the method previously described^38^.

### FEM simulation for contact pressure analysis

In order to estimate the contact pressure at LM, FEM analysis was performed using ABAQUS to investigate the effect of the LMs of LMDEB on the contact pressure between LMDEB and the luminal surface of vascular tissue. The finite element model was assumed to be a plane strain state since the longitudinal length is relatively longer than the diameter of LMDEB. The material properties such as the modulus of elasticity and the Poisson’s ratio of LMDEB and the vascular tissue of rabbit femoral artery were 500 MPa, 0.1 and 3.50 MPa, 0.3, respectively. The finite element model has sufficiently refined meshes that have 20 contact nodes in any contact region. The model has 52,627 plane strain elements, with a total of 57,071 nodes (Fig. 5B). The contact condition between LMDEB and artery was assumed to be frictionless. A displacement was applied to the compressed LMDEB in the radial direction and is gradually increased until LMDEB was fully expanded to the diameter of 2.75 mm, as shown in Fig. 5A.

### *In vivo* tests with rabbit model

A total of 26 male New Zealand White rabbits (3.5–4.0 kg weight) were used in this study (12 rabbits for the drug distribution test. and 14 rabbits for the efficacy test in Section 3.6). All rabbits were acclimatized for a week, housed at room temperature with a 12-hour cycle with free access to standard diet and water. The study protocol was approved by the local Institutional Animal Care and Use Committee of Yonsei University Health System (IACUC: 2015–0028 and Cardiovascular Production Evaluation Center, Yonsei University College of Medicine). All animals received human care in compliance with the Animal Welfare Act and the “Principles of Laboratory Animal Care” formulated by the Institute of Laboratory Animal Resources (National Research Council, NIH Publication No. 85–23, revised 1996).

After premedication with antibiotics and analgesics, anesthesia was induced by intramuscular injection with an appropriate Zoletil (10 mg/kg, Virvac, USA) and Rompun (5 mg/kg, Bayer, Germany), then maintained with 2–3% of isoflurane (Forane®, JW Pharm, Republic of Korea) and oxygen. Access to the iliac artery was obtained via the carotid artery, using a sterile surgical technique. Heparin (150 units/kg) was injected to maintain an activated clotting time within 250 seconds before catheterization. Rabbits for atherosclerosis model were injured with inflated balloons after fed a high cholesterol diet (1% cholesterol, DooYeol Biotech, Republic of Korea). During the intervention procedure, oversized balloon inflation with 1.3:1.0 balloon-artery ratio was performed one time for 30 seconds within both iliac arteries (right and left). Then, conventional DEB (right iliac) (Genoss Co., Ltd., South Korea) and LMDEB (left iliac) were applied to the vessels for 60 seconds at iliac arteries. In the experiment to investigate drug distribution and efficiency of LMDEB, 14 normal rabbits were used with both LMDEB and conventional DEB coated either RB or PTX + FP as a model drug. The balloon inflation conditions are the same as before. All rabbits received aspirin (40 mg/kg) and clopidogrel (75 mg/daily) at the end of the procedure.

### *In vivo* tests with ISR minipig model

A total of 2 male minipigs, aged 6 months, weighing 35 kg were used in the study. The study protocol was also approved by Yonsei University’s IACUC (2016–0028) as described previously. All the pigs were given antiplatelet pretreatment with aspirin (10 mg/kg) and clopidogrel (75 mg/daily) as well as antibiotics and analgesics. Anesthesia was induced by intramuscular injection with an appropriate mixture of Zoletil and Rompun, then maintained with 1.5% of isoflurane and oxygen. Heparin (200 units/kg) was injected to maintain an activated clotting time within 250 seconds before catheterization.

Two right coronary arteries (RCA), left anterior descending arteries (LAD) and left circumflex arteries (LCX), respectively, total 6 coronary arteries of 2 minipigs were blinded to the randomization assignment and divided into 2 groups; conventional DEB (n = 3) and LMDEB (n = 3). Access to the coronary artery was obtained via the carotid artery using the sterile surgical technique. Oversized balloon inflation with a 1.3:1.0 balloon-artery ratio was repeated twice for 30 seconds each, followed by implantation of a 3 mm (length) × 18 mm (diameter) stent in each major coronary artery. We reconfirmed balloon inflation pressure to achieve the desired overstretching by angiography. 4 weeks after the procedure we confirmed ISR formation in the minipig model by OCT and QCA analysis. These two ISR induced minipigs were treated by balloon inflation for 60 seconds with conventional DEBs or LMDEBs coated with PTX by following the same procedure as described above in the *in vivo* tests with rabbit models. After 4 weeks, OCT and QCA were performed, and euthanasia was carried out to harvest coronary arteries.

### Fluorescence and histologic image analysis

For fluorescent analysis to visualize drug distribution, target vessels were cryo-sectioned. Harvested tissue samples were immediately embedded in optimum cutting temperature compound (Tissue-Tek, Sakuta Finetek, USA) and frozen with dry ice. Frozen samples were sectioned with a microtome at 10 µm thickness, and then images were taken using a fluorescent microscope (BX53, Olympus, Japan) under identical imaging condition (light intensity = 6, exposure time = 1 second, sensitivity = ISO 100). The images were analyzed with an image analyzer program (DP Controller, Olympus, Japan). Afterwards, the fluorescent intensities of these sample images were calculated by measuring the ID as described previously. Since the sectional area of the blood vessel varies from sample to sample, the measured value was normalized by the area of the blood vessel to obtain normalized integrated density (NID).

For therapeutic efficacy analysis, target vessels were paraffinized and stained. Cultured vascular samples were fixed by continued perfusion with 10% normal buffered formalin for a day. For paraffin embedding, a 5 mm long sample of the vessel was placed intactly into a single cassette and processed through a graded series of alcohols and xylenes, and then the specimens were embedded in a single paraffin block. Paraffinized samples were sectioned with a microtome at 4 µm thickness, mounted on silane-coated slides, and stained with hematoxylin and eosin (H&E) (Merck, Darmstadt, Germany), Masson’s trichrome (BBC Biochemical, USA). The cross-sectional area was measured using an optical microscope (SCN400, Leica, Germany) and histomorphometry was performed using LAS 4.2 software.

### LS/MS measurement of drug residue in vascular tissue

The target vascular lesions treated with DEB or LMDEB were harvested after 2 days by sacrificing the rabbits with potassium injection. The harvested tissue samples soaked in 200 µl mixture of methanol and acetonitrile (J.T. Baker® Chemicals, USA) (w/w = 1:1) were pulverized using a sonicator (Q500, Qsonica, USA) and mixed with a mixing block (MB-102, BioER, China) for 30 minutes. A standard curve was prepared at 0.01, 0.1, 0.5, 1, and 5 ppm. All sample aliquots were transferred into autosampler vials at 10 µl. The PTX concentrations of the homogenates were quantified through an LC/MS system (Agilent 6530 Accurate-Mass Q-TOF LC/MS, Agilent Technologies, USA). The flow rate of LC/MS was 0.5 ml/min through a column of extend C-18 (Agilent Technologies, USA). The mobile phase was the mixture of methanol and acetonitrile (w/w = 1:1). PTX in the aliquots was detected by the mass spectrometry in multiple reaction-monitoring modes with a transition of PTX from 876.3218 AMU.

### Vascular stenosis evaluation

The degree of stenosis was assessed over the entire 10 mm length of treated vessel following DEB and LMDEB intervention. QCA and OCT were performed immediately after balloon inflation and after 4 weeks to monitor the progress of intimal formation in terms of DS and AS. OCT was operated using a C7-XR imaging system (LightLab Imaging Inc., St. Jude Medical, USA). Contrast media was constantly flushed at a flow rate of 4 mL/s through a guiding catheter for 4 seconds. The OCT catheter was pulled back at a rate of 20 mm/s, and the OCT image acquisition frequency was 100 frames/s. All OCT images were analyzed at an independent core laboratory (Cardiovascular Research Center, Republic of Korea) by analysts blinded to procedural information. The histological images were compared to the proximity to side branches, and the length from the balloon injury edge to the location of the histology samples (provided by the histological laboratory).

## Electronic supplementary material

Supplementary information
