# Supplementary material for: Linear Micro-patterned Drug Eluting Balloon (LMDEB) for Enhanced Endovascular Drug Delivery
Source: Sci Rep. 2018 Mar 5;8:3666. doi: 10.1038/s41598-018-21649-7 (PMC5838243; doi:10.1038/s41598-018-21649-7)
Supplement: Supplementary file 1 — Supplementary information [file 41598_2018_21649_MOESM1_ESM.doc]

Supporting Information

Linear Micro-patterned Drug Eluting Balloon (LMDEB) for Enhanced Endovascular Drug Delivery

Kang Ju Lee^1^, Seul Gee Lee^2^, Ilkwang Jang^1^, Seung Hyun Park^1^, Da Som Yang^1^, Il Ho Seo^1^, Sung-Kyung Bong^2^, Duk Hwan An^3^, Min Kwon Lee^3^, In Kwon Jung^3^, Yong Hoon Jang^1^, Jung Sun Kim^4^*, WonHyoung Ryu^1^*

**Author Address**

^1^School of Mechanical Engineering, Yonsei University, 50 Yonsei-ro, Seodaemun-gu, Seoul 03722, Republic of Korea

^2^Graduate Program in Science for Aging, Yonsei University, 50 Yonsei-ro, Seodaemun-gu, Seoul, 03722, Republic of Korea

^3^Genoss Co., Ltd., 1F, Gyeonggi R&DB center / 226 2F GSBC, 105 Gwanggyo-ro, Yeongtong-gu, Suwon-si, 16229, Republic of Korea

^4^Division of Cardiology, Severance Cardiovascular Hospital, Yonsei University College of Medicine, 50 Yonsei-ro, Seodaemun-gu, Seoul, 03722, Republic of Korea

Corresponding Authors

**Jung Sun Kim**, Division of Cardiology, Severance Cardiovascular Hospital, Yonsei University College of Medicine, 50 Yonsei-ro, Seodaemun-gu, Seoul, 03722, Republic of Korea. Phone: 82-2-2228-8457; E-mail: kjs1218@yuhs.ac

**WonHyoung Ryu**, Department of Mechanical Engineering, Yonsei University, 50 Yonsei-ro, Seodaemun-gu, Seoul, 03722, Republic of Korea. Phone: 82-2-2123-5821; Fax: 82-2-312-2159; E-mail: whryu@yonsei.ac.kr

***<*Paraffin-fixation vs. cryo-sectioning*>***

There is no significant difference between LMDEB and DEB from paraffin slides under 3 seconds light exposure time. Clear difference was shown in cryo-sectioned slides under only 1 second light exposure time. Since it takes at least 24 hours to prepare paraffin block samples, drug molecules such as RB or PTX continuously diffuse during the preparation. This makes it difficult to analyze the ‘original’ distribution of the drug molecules in the tissue samples. When the delivered amount of drug is relatively small, then the difference of the delivered drug in the tissue samples becomes negligible due to the diffusion during paraffin preparation. On the other hand, the cryo-sectioned samples provide more ‘accurate original’ distribution of drug delivered tissue, since the tissue samples are frozen for 15 seconds.


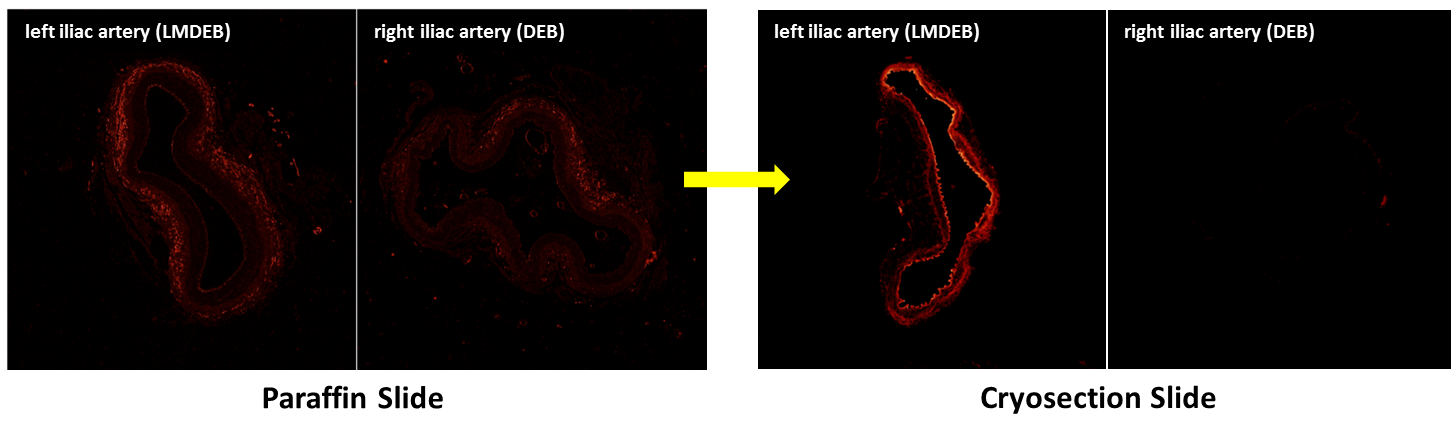


Figure S1. Comparison of fluorescent images between paraffin-fixed and cryo-sectioned slides.

*<*Calculation of I/P, DS, and AS in Atherosclerotic Rabbit Model*>*


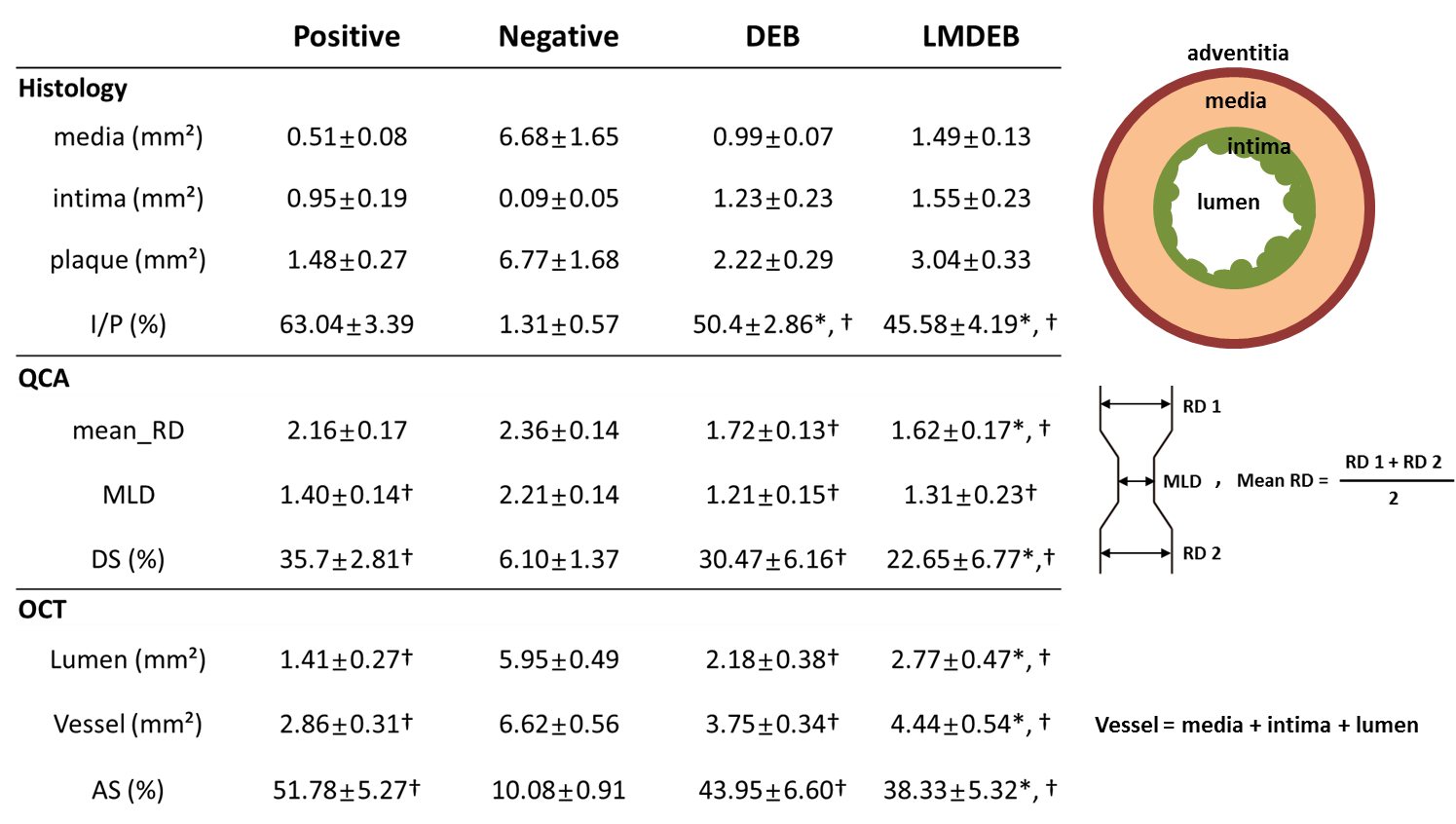


Figure S2. Experimentally-measured values for calculating intima-plaque ratios (I/P), diameter stenosis (DS) and area stenosis (AS). All data were presented as the mean±SEM. *p < 0.05, compared with positive group. †p < 0.05, compared with negative group.

*<*Calculation of DS, and AS in ISR Minipig Model*>*


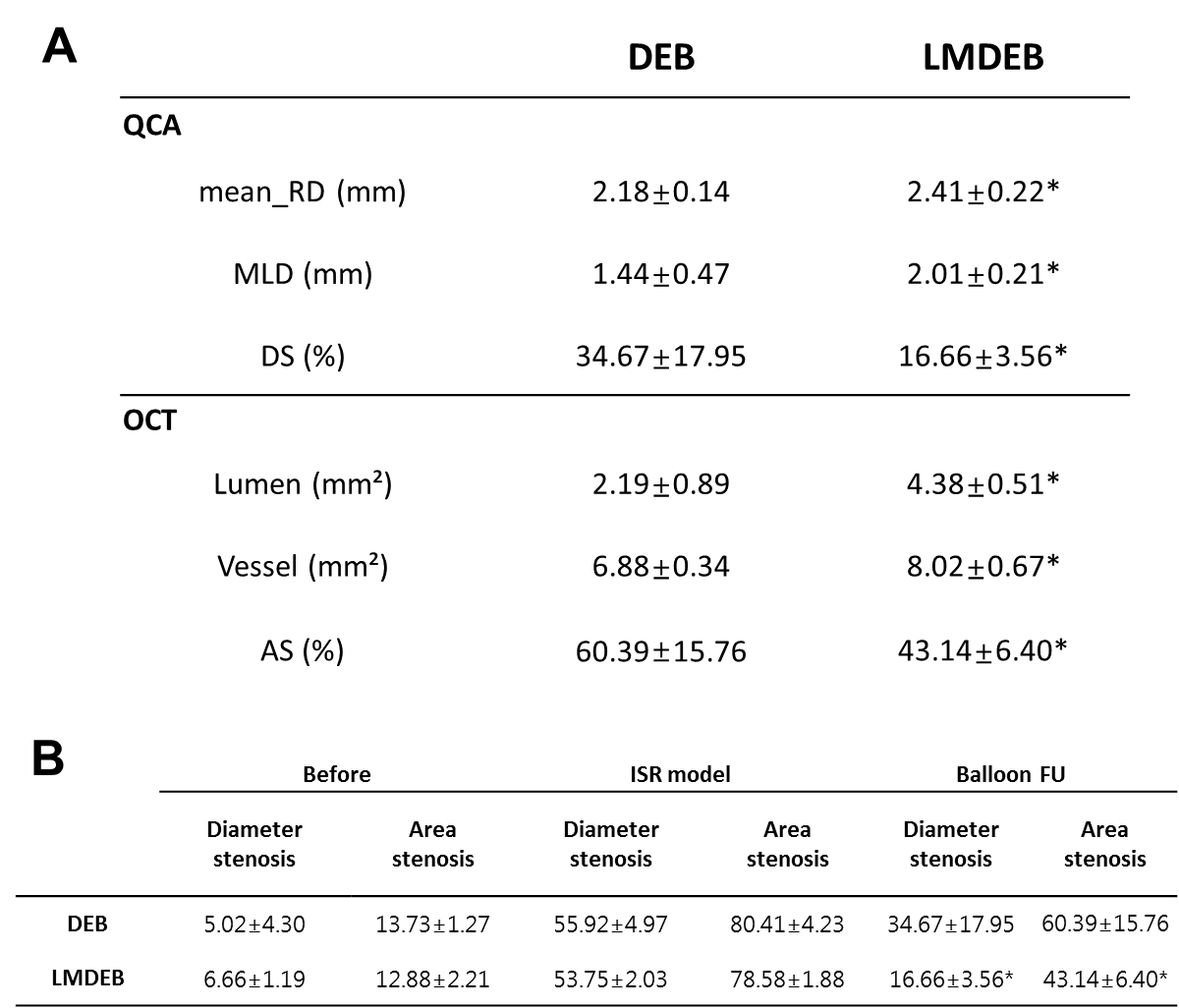


Figure S3. (A) Experimentally-measured values for diameter stenosis (DS) and area stenosis (AS). (B) Detailed values of data in Figure 10. All data were presented as the mean±SEM. *p < 0.05, compared with DEB group.
